# Gut microbiota signatures in mild cognitive impairment: a scoping review of microbial taxa and clinical implications

**DOI:** 10.3389/fmicb.2026.1895237

**Published:** 2026-09-17

**Authors:** Óscar Gabriel Beltrán, Andrea Angarita-Rodríguez, Janneth González, Edgar D. Hernández, Andrés Pinzón

**Affiliations:** 1 Laboratorio de Bioinformática y Biología de Sistemas, Universidad Nacional de Colombia Bogotá, Bogotá, Colombia; 2 Departamento de Nutrición y Bioquímica, Facultad de Ciencias, Pontificia Universidad Javeriana, Bogotá, Colombia; 3 Facultad de Medicina, Departamento de Movimiento Corporal Humano, Maestría en Fisioterapia del Deporte y la Actividad Física, Universidad Nacional de Colombia Bogotá, Bogotá, Colombia

**Keywords:** Alzheimer’s disease, biomarkers, dysbiosis, gut microbiota, gut-brain axis, mild cognitive impairment, neuroinflammation, scoping review

## Abstract

**Objective:**

To identify systematically the typical gut microbiota in mild cognitive impairment patients and its association in disease progression. Systematically identify microbial taxa consistently associated with Mild Cognitive Impairment.

**Design:**

A scoping review was performed according to the guidelines of the Joanna Briggs Institute using CoCoPop design.

**Study selection:**

Several studies about gut microbiota sequencing in mild cognitive impairment diagnosed patients compared against healthy controls.

**Methods:**

Searches in PubMed, Embase, Scopus, and Web of Science using MeSH and DeCS terms, were performed up to December 2025. Two reviewers independently screened titles and abstracts. Then, the full texts were reviewed to select documents to be included. When there were disagreements in papers selection, those were resolved after discussions among the reviewers. Relevant data information from selected publications was extracted, and a descriptive analysis was made.

**Results:**

The documents do not show drastic changes in the alpha or beta diversity of the microbiota, but they do identify consistent alterations in the relative abundance of specific taxa that allow patients with MCI to be distinguished from healthy controls. Among the most relevant findings is the decrease in SCFA-producing genera, such as *Ruminococcus* and *Lachnospira*, whose reduction is directly associated with the deterioration of key brain functions, including synaptic processes and neuronal oxidative metabolism. On the other hand, there is an increase in proinflammatory taxa such as Bacteroides, a pattern that is similar to that observed in patients with Alzheimer’s disease, although generally less pronounced.

**Conclusion:**

The results highlight that alterations in the abundance of specific bacteria, characterized by the loss of protective taxa and an increase in proinflammatory bacteria, serve as early biomarkers for detecting and predicting Mild Cognitive Impairment (MCI). This pattern of proinflammatory dysbiosis can be identified even in preclinical stages, and it has been proposed as a potential noninvasive tool for early diagnosis and therapeutic intervention before dementia progresses.

## 1 Introduction

Mild Cognitive Impairment (MCI) is a clinical condition considered as a transitional stage between normal cognition and dementia, mainly Alzheimer disease (AD) (Cabrera et al., 2025; Nagpal et al., 2020; Zhu et al., 2022), although it is also considered a non-motor symptom of Parkinson disease (PD) (Aho et al., 2024; Auclair-Ouellet et al., 2025). It is characterized by cognitive decline greater than expected for age, without significantly interfering with daily functional abilities (Nagpal et al., 2020; Zhang et al., 2021). The prevalence of MCI among individuals over 60 years ranges from 5 to 36.7% with an annual progression rate to dementia of 5 to 39% (Escarcega et al., 2025; Ma, 2020). However, this progression is heterogeneous and influenced by various factors including MCI subtypes, follow-up duration, and individual biological characteristics such as APOE genotype (Escarcega et al., 2025; Ma, 2020; Zhu et al., 2022).

Diagnosis of MCI involves a multistep clinical approach integrating clinical history assessment, neuropsychological tests, and sometimes, neuroimages and biomarker analysis (Ma, 2020; Zhu et al., 2022). Standardized tests such as the Mini-Mental State Examination (MMSE) and the Montreal Cognitive Assessment (MoCA) are widely used to detect early cognitive alterations, particularly in domains such as memory, language, and executive function (Li et al., 2023; Zhang et al., 2021; Zhu et al., 2022).

In recent years, increasing attention has been given to the gut microbiota as a modifiable factor associated with brain health. The gut microbiota, comprising millions of microorganisms residing in the human gastrointestinal tract, communicate bidirectionally with the central nervous system through the gut-microbiota-brain axis (Afzaal et al., 2022; Li et al., 2023). This complex network involves neural, immune, endocrine, and metabolic signaling (Afzaal et al., 2022; Li et al., 2023; Liang et al., 2022; Wanapaisan et al., 2023). Evidence suggests that alterations in gut microbial composition can influence cognitive function, neuroinflammation, and even the structural integrity of the brain (Liang et al., 2022; Wanapaisan et al., 2023). Although some studies have reported no significant differences in alpha nor beta diversity between MCI patients and cognitively normal controls (Aho et al., 2024; Cabrera et al., 2025; Sheng et al., 2021; Zhu et al., 2022), others have consistently observed taxonomic shifts in specific bacterial genera (e.g., reduced *Faecalibacterium, Roseburia*; increased *Lactobacillus, Akkermansia*) across diverse populations and study designs (Li et al., 2024; Li et al., 2023; Sheng et al., 2021; Wanapaisan et al., 2023). These microbial alterations are particularly relevant because many of the affected taxa are involved in short-chain fatty acid (SCFA) production or modulation of intestinal permeability—both of which have been linked to neuroinflammation and cognitive decline (Boschetti et al., 2023; Li et al., 2023; Wanapaisan et al., 2023).

Taken together, these findings support the hypothesis that specific gut microbial patterns may serve as early indicators or modulators of MCI progression. This review aims to systematically identify differentially abundant bacterial taxa between MCI patients and cognitively healthy controls and to assess their potential as microbial biomarkers for disease monitoring and intervention strategies.

## 2 Methods

To identify relevant studies, a scoping review was conducted following the guidelines of the Joanna Briggs Institute, based on a Condition, Context, Population (CoCoPop) question format (Boschetti et al., 2023; Khalil et al., 2019; Munn et al., 2018; Peters et al., 2015, 2020). Additionally, the guidelines Preferred Reporting Items for Systematic reviews and Meta-Analyses extension for Scoping Reviews (PRISMA-ScR) was followed (Tricco et al., 2018).

### 2.1 Search strategy

The search aimed to identify publications that report the gut microbial composition of healthy and mild cognitive impairment adults. The search strategy was developed and refined through an iterative calibration process (Peters et al., 2020). Exploratory searches were conducted to identify commonly used terms and keywords, while evaluating the ability of the search strategy to retrieve previously identified reference studies. Relevant publications were searched in PubMed, Embase, Scopus, and Web of Science, in a first round from January 2021 to February 2025 and an update from February 2025 to December 2025, using main descriptors indexed in MeSH and DeCS databases. The structure of the search strategy were adapted to the interface specifications for Boolean operators and wildcards of each one database.

The search terms were (human) AND ((Gastric OR Gastrointestinal OR Gut OR Intestinal) AND (Microbiome OR Microbiota)) AND ((Mild Cognitive Impairment) OR ((Cognitive) AND (Decline OR Disorder OR Dysfunction OR Impairment))) AND (Gut Brain Axis OR Gut Brain Microbiome Axis OR Gut-Brain Axis OR Microbiome Brain Gut Axis OR Microbiota Gut Brain Axis NOT (skin Microbiome OR cancer OR vagina OR dementia OR epilepsy OR COVID OR diabetes OR psychiatric)). Additionally, cross-referencing of the included studies was performed to identify relevant articles not retrieved through the electronic search. All eligible documents were processed in Rayyan platform (Qatar Computing Research Institute) (Ouzzani et al., 2016).

### 2.2 Study selection

#### 2.2.1 Inclusion criteria

Observational analysis of the human gut microbiota of individuals diagnosed with mild cognitive impairment and healthy controls, the subjects included were adults older than 18 years, publication is less than 6 years (2020–2025), full text publication should be in English or Spanish, and available for free download.

#### 2.2.2 Exclusion criteria

(1) The publication promotes any available or new drug and/or treatment
(2) Publications produced primarily for commercial, marketing, or promotional purposes rather than reporting original peer-reviewed observational research were excluded.
(3) Use of probiotics and/or dietary supplement
(4) Use of antibiotics
(5) Pregnant and/or lactating women
(6) Alcoholism
(7) Encephalopathy (miscellaneous)
(8) Autoimmunities
(9)Neurological disorders
(10) Consumption of anti-psychotic and anti-convulsive drugs
(11) History of cranio-encephalic trauma
(12) Post-surgical effects studies
(13) Gastrointestinal cancer
(14) Chemotherapy
(15) Hepatic cirrhosis
(16) Proton pump inhibitors
(17) Microbiota transplantation
(18) HIV
(19) Organ transplantation

### 2.3 Data charting

A spreadsheet-based data extraction matrix was developed in LibreOffice v24.2.2.7.2 to support the data charting process and was refined based on previously described recommendations (Peters et al., 2015, 2020). The characteristics of interests were extracted: title, authors, year, country, sample size, sequencing technology, bacterial taxa, and findings of microbial diversity. The complete data extraction matrix for all included studies is provided in Supplementary Table S1.

### 2.4 Data analysis

After removing duplicates, two reviewers independently screened the titles and abstracts according to the predefined eligibility criteria. Studies considered potentially eligible were retrieved, and their full texts were independently assessed by the same reviewers for final inclusion. When disagreements arose regarding study selection, a third reviewer independently assessed the article. The final decision was reached through discussion and consensus among the three reviewers (Peters et al., 2020). In cases of interesting publications referenced by the selected articles, these were reviewed as cross-references (Peters et al., 2015). Although critical appraisal is not mandatory in scoping reviews, it was undertaken to provide an overview of the methodological quality of the included prevalence studies and to facilitate interpretation of the analyzed evidence Supplementary Table S2 (Munn et al., 2015). The information is presented in a narrative format, accompanied by tables that summarize it.

The names, taxonomic definitions, and phenotypic characteristics of the taxa of interest were manually verified in the List of Prokaryotic Names with Standing in Nomenclature (LPSN) (Göker et al., 2025), GTDB (Chaumeil et al., 2022), and BacDive (Schober et al., 2025).

## 3 Results

The studies selection process is presented in Figure 1. From the electronic database search, 753 articles published in scientific journals and four cross-referencing studies were located initially of which 81 duplicated publications were removed. Subsequently, 646 were excluded for the title and abstract. Of the 29 studies read in full text, 19 articles were excluded. Finally, nine publications were included.

**Figure 1:**
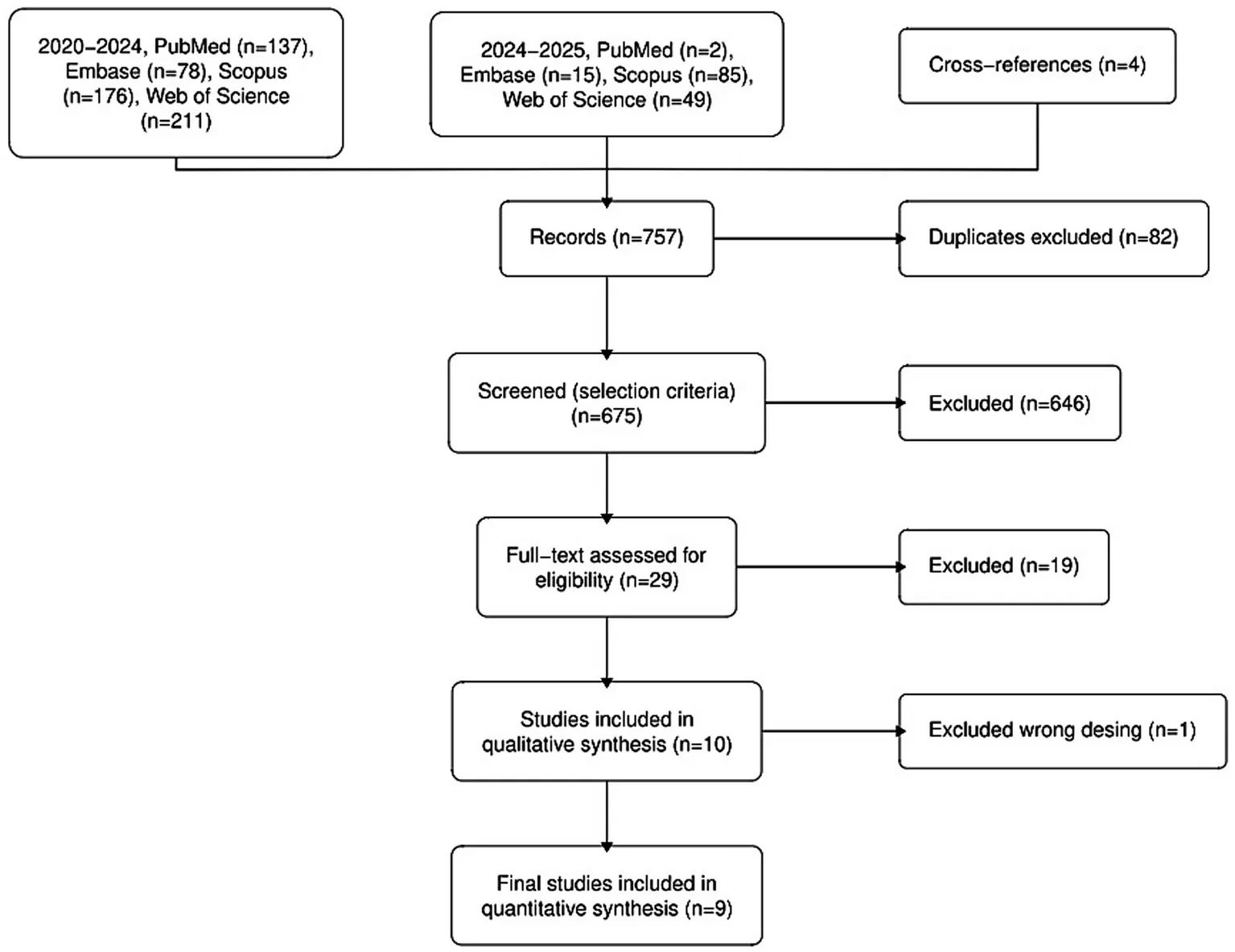
Flow chart of the evidence search and selection process.

After removing duplicates, title and abstract screening was conducted according to eligibility criteria by two reviewers. Then, pre-selected papers were retrieved and full-text read, in cases where there were papers disagreements selection, a third one reviewer checked the conflicts and the disagreements were resolved after discussions among the reviewers. In cases of interesting publications referenced by the selected articles, these were reviewed as cross-references. An extraction matrix was created to retrieve relevant data from included publications. Then, data extracted included information about study design, year and place of publication, sequencing technology, patient conditions, and identified bacterial taxa.

The names, taxonomic definitions, and phenotypic characteristics of the taxa of interest were manually verified in the List of Prokaryotic Names with Standing in Nomenclature (LPSN) (Göker et al., 2025), GTDB (Chaumeil et al., 2022), and BacDive (Schober et al., 2025).

### 3.1 Study data

The included studies (case–control) come from Asia, Europe, and USA in countries such as Albania, China, India, South Korea, Taiwan, and USA published between 2019 and 2025, and they were cross-sectional studies that included 431 cases and 586 healthy controls. Groups sizes range from 20 to 119 patients with MCI, along with control groups of 18 to 320 individuals (Table 1) This geographic and population diversity reflects different diets and environmental contexts that may modulate the microbiota, although it also introduces heterogeneity into the findings. The main analysis included diversity analyses such as alpha and beta diversity expressed as Jaccard, Shannon, Bray-Curtis, unweighted Unifrac, and weighted Unifrac indexes and taxonomic composition assessment.

**Table 1:** Basic characteristics of the included studies.

Reference	year	Country	Cases	Controls	Main findings
Zhao et al. (2025)	2025	China	75	61	It identifies pathways where the microbiota influences fecal metabolites (such as arachidonic acid), which in turn impact brain atrophy and cognition.
Fan et al. (2025)	2025	Taiwan	119	320	It identifies 59 important species and demonstrates that species of the same genus can have opposite effects on the risk of MCI and amyloid pathology.
Troci et al. (2024)	2024	Germany	84	50	It describes changes in oral and fecal communities from prodromal stages; it highlights *Faecalibacterium* as the basis of homeostasis in healthy subjects and an increase in Gram-negative oral pathogens correlated with biomarkers in CSF.
Kim et al. (2023)	2023	South Korea	40	40	It identifies significant differences in specific genera (*Bacteroides, Prevotella, Akkermansia*) and a marked reduction in amino acid biosynthesis pathways (tryptophan) linked to patients with MCI.
Hung et al. (2023)	2023	Taiwan	20	20	It demonstrates that the reduction of the *Ruminococcus* genus is associated with affective symptoms (anxiety/depression) in aMCI+ patients.
Chaudhari et al. (2023)	2023	USA	23	25	Identifying different “trans-kingdom” forms, with a decrease in bacterial abundance along with an increase in viruses, the combination of which improves the prediction of cognitive decline.
Guo et al. (2021)	2021	China	20	18	It proposes that in patients with MCI, dysbiosis worsens with the stage of the disease and highlights *Lachnospira* as the only genus whose reduction was statistically significant specifically in the MCI group.
Liu et al. (2021)	2019	China	20	22	Discover specific interaction patterns where Bacteroides abundance is negatively correlated with spontaneous intrinsic brain activity (fALFF) I fractional amplitude of low-frequency fluctuations (fALFF) in aMCI patients.
Li et al. (2019)	2019	China	30	30	It establishes that the microbiota of patients with MCI presents alterations similar to those of patients with Alzheimer’s disease, which demonstrates that dysbiosis occurs years before dementia; fecal matter samples allowed for the correct identification of 93% of patients with MCI.

### 3.2 Clinical and demographic importance

The gut microbiota composition varies according to gender, country, and ethnicity (Chaudhari et al., 2023; Kim et al., 2023; Troci et al., 2024). Some background have shown that microorganisms with significant differences and disease-associated taxa in MCI can vary in geographically distinct study populations, even within the close Asian population (Guo et al., 2021; Kim et al., 2023; Troci et al., 2024).

Recent studies suggest that MCI and AD are associated with complex gut microbiome dysbiosis (Guo et al., 2021; Kim et al., 2023; Li et al., 2019). These microbial patterns may vary across populations, underscoring the importance of race and geography-specific research and the need for large-scale longitudinal studies to validate these findings and develop targeted biomarkers and therapies (Guo et al., 2021; Kim et al., 2023; Li et al., 2019; Liu et al., 2021). Therefore, to characterize the changes in the abundance of key bacteria may be useful for early diagnosis and prognosis for these pathologies (Chaudhari et al., 2023; Li et al., 2019; Liu et al., 2021).

### 3.3 Microbial diversity

Although some studies reported no differences in alpha or beta diversity between MCI and healthy controls, some changes were observed in the relative abundance of specific bacterial taxa (Table 2). In contrast, other studies have reported a slight diversity decrease in gut microbiota of patients with AD due to several differences between study populations (Troci et al., 2024). In fact, it has been reported that lower alpha diversity in bacteria and higher virus abundance in the gut of subjects with MCI compared against healthy controls, although bacterial alpha diversity did not reach statistical significance (Chaudhari et al., 2023).

**Table 2:** List of important bacteria and their potential impact.

Bacteria	Category	Function	Clinic effect	Reference
*Lachnospira, Faecalibacterium, Roseburia, Anaerostipes*	SCFA production and neuroprotection	Production of SCFA such as butyrate and propionate. These maintain the blood–brain barrier and reduce amyloid deposition.	Its reduction correlates positively with low scores in MMSE and MoCA. The loss of these microbes predicts decline in memory and executive functions.	Chaudhari et al. (2023), Guo et al. (2021), Kim et al. (2023), Troci et al. (2024), and Zhao et al. (2025)
*Ruminococcus, Bacteroides eggerthii*	Neurotransmitter metabolism and affective health	They participate in the metabolism of tryptophan for the biosynthesis of serotonin and tryptamine. Tryptamine acts as a neuromodulator of brain activity.	Its decrease is linked to affective symptoms such as anxiety and depression.	Fan et al. (2025) and Hung et al. (2023)
*Akkermansia muciniphila, Prevotella*	Intestinal barrier integrity and homeostasis	They stimulate the formation of the mucus layer and maintain intestinal integrity and the epithelial barrier. They prevent the translocation of bacterial products such as toxins and LPS that cause neuroinflammation.	The reduction of these microorganisms facilitates systemic neuroinflammation, accelerating brain atrophy and the deterioration of recognition memory.	Fan et al. (2025) and Kim et al. (2023)
*Lactococcus lactis, Clostridium symbiosum*	Energy metabolism	Production of lactic acid to protect mitochondria from amyloid damage. Regulation of NAD + and branched-chain amino acid (BCAA) metabolism.	The reduction of these functions affects neuronal oxidative metabolism and is associated with a decrease in hippocampal volume.	Fan et al. (2025) and Hung et al. (2023)

Multiple studies suggest that alterations in the MCI gut microbiome are similar to those in AD patients, although often less severe (Guo et al., 2021; Li et al., 2019; Troci et al., 2024). For instance, the alterations in abundance of *Bacteroides, Faecalibacterium,* and *Prevotella* had the same direction in MCI and AD patients, although the changes were only significant in AD (Guo et al., 2021; Troci et al., 2024). In the same way, in a Korean study (Kim et al., 2023), no significant difference in the overall microbial profile was observed between the MCI and health control patients, suggesting that changes occur in the relative abundance of specific microbes. The results on diversity show that, although some studies report no changes in alpha or beta diversity (Hung et al., 2023; Kim et al., 2023; Liu et al., 2021), others document decreased bacterial richness (whole tree PD) and alterations in overall structure (Guo et al., 2021; Li et al., 2019). These differences suggest that, although MCI is not always accompanied by drastic changes in diversity, the microbiota in some patients shows less resilience.

Relative composition shows increases in *Bacteroides, Parabacteroides, Akkermansia*, and *Prevotella* in certain cases (Kim et al., 2023; Troci et al., 2024), while others report reductions in the same genera (Guo et al., 2021; Li et al., 2019). More consistently, a loss of short-chain fatty acid-producing bacteria such as *Ruminococcus, Lactococcus*, and *Lachnospira* is detected (Fan et al., 2025; Guo et al., 2021; Hung et al., 2023) (Figure 2). Furthermore, some studies identify variations in phage presence and transkingdom interactions within the microbiome (Chaudhari et al., 2023).

**Figure 2:**
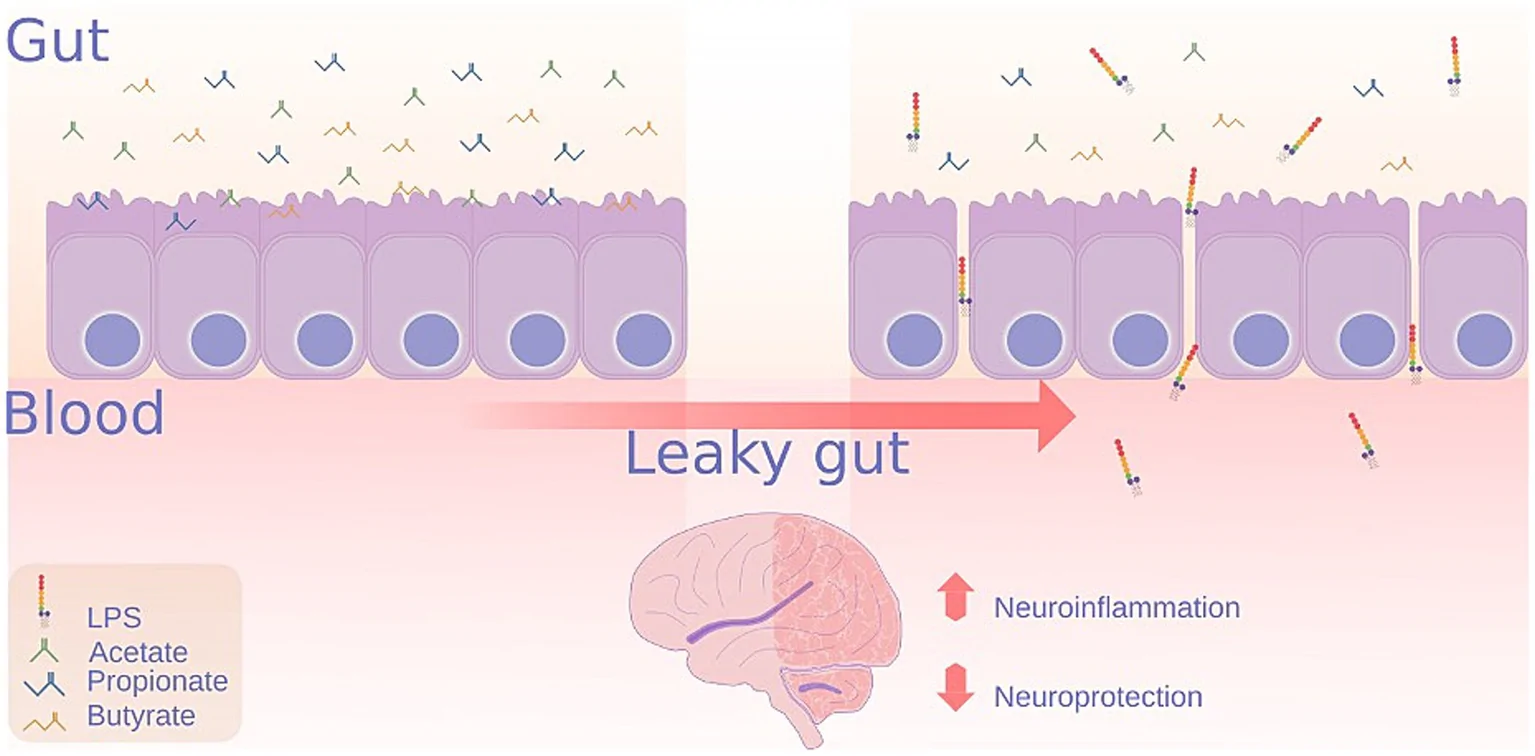
In patients with mild cognitive impairment (MCI), the loss of protective bacteria reduces acetate and propionate levels, while the increase in pro-inflammatory taxa raises LPS levels. This, facilitated by a leaky gut, results in systemic inflammation that directly affects synaptic function and neuronal oxidative metabolism.

### 3.4 Decreased bacteria

#### 3.4.1 *Ruminococcus* and *Lactococcus*

Significantly decreased in amyloid-positive amnestic mild cognitive impairment (aMCI+) patients compared to healthy controls (Hung et al., 2023). *Ruminococcus*, which belongs to the phylum *Firmicutes* and is a propionate producer, has been found as decreased in MCI/dementia patients (Hung et al., 2023), and this reduction is negatively correlated with affective symptoms in aMCI+ (Hung et al., 2023). Also, the abundance reduction affects tryptophan metabolism for tryptamine and serotonin biosynthesis promoting affective symptoms and cingulate white matter degradation (Hung et al., 2023). The abundance reduction of *Lactococcus,* which participates in the production of L-lactic acid and activates mucosal function and immunity; can lead to impairments in brain functions, including a decrease in synaptic process, axonal excitability, and neural oxidative metabolism (Chaudhari et al., 2023; Hung et al., 2023; Troci et al., 2024).

#### 3.4.2 *Prevotella* and *Akkermansia*

There is evidence about *Prevotella* relative abundance reduction in Korean MCI patients, which correlates negatively with the abundance of *Bacteroide*s (Guo et al., 2021; Kim et al., 2023). However, it contrasts with Chinese results where *Prevotella* abundance was higher in MCI patients (Guo et al., 2021). These discrepancies highlight the heterogeneity of gut microbiota dysbiosis in cognitive impairment that is associated with several factors such as diet, genetics, and lifestyles (Guo et al., 2021; Kim et al., 2023). In the same way, *Akkermansia* is considered a beneficial bacterium, because it helps to keep the epithelial cell layer integrity and modulates the immune system by producing propionate (Bedarf et al., 2017; Cantu-Jungles et al., 2025; Kim et al., 2023). Therefore, its reduction is associated with intestinal barrier dysfunction, a risk factor for AD, as it has been reported in MCI Korean patients (Kim et al., 2023). The abundance reduction of *Prevotella* and *Akkermansia* affects mucus production, allowing lipopolysaccharides (LPS) and bacterial amyloids to cross into the blood and brain, triggering neuroinflammation (Kim et al., 2023; Troci et al., 2024).

#### 3.4.3 Bacteroides

This genus has been positively correlated with p-tau and p-tau/Aβ42 proteins, which are recognized as AD markers (Fan et al., 2025; Kim et al., 2023; Troci et al., 2024). The *Bacteroides* abundance increase may lead to greater translocation of lipopolysaccharides (LPS) from the gut to the bloodstream and brain, contributing to AD pathology (Kim et al., 2023; Li et al., 2024). For instance, there have been reported an increase in *Bacteroides* abundance in Korean MCI patients (Kim et al., 2023). In contrast, the abundance was lower in Chinese people (Guo et al., 2021). Nevertheless, some *Bacteroides* species have beneficial effects such as *B. eggerthii* (Fan et al., 2025)*. This causes* the role of *Bacteroides* genus to vary depending on the different species involved.

#### 3.4.4 Short-chain fatty acids (SCFA) reduction

SCFAs produced by the gut microbiota (acetate, propionate, and butyrate) play a key role in gut–brain communication, exert neuroprotective effects, maintain intestinal barrier integrity, and reduces amyloid deposition in the brain (Chaudhari et al., 2023; Guo et al., 2021). However, a reduction in butyrate-producing bacteria has been consistently reported in patients with mild cognitive impairment (MCI), with potential consequences for intestinal barrier integrity and inflammatory regulation (Chaudhari et al., 2023). Members of the *Lachnospiraceae* family, the genus *Subdoligranulum*, and the species *Roseburia intestinalis* and *Roseburia hominis* are major butyrate producers that have demonstrated protective effects against cognitive decline; nevertheless, their abundance is reduced in individuals with MCI (Chaudhari et al., 2023; Fan et al., 2025; Guo et al., 2021). Similarly, decreased levels of butyrate-producing bacteria have been reported in AD and PD (Forero-Rodríguez et al., 2024; Nuzum et al., 2023; Wanapaisan et al., 2023). The loss of these bacteria is associated with a proinflammatory environment, which may contribute to the progression from MCI to dementia (Chaudhari et al., 2023). In addition, propionate production is reduced as a consequence of decreased abundance of genera such as *Akkermansia* and *Ruminococcus* (Hung et al., 2023; Kim et al., 2023). Likewise, a decrease in *Akkermansia*, *Blautia*, and *Ruminococcus* has been associated with reduced acetate levels. Acetate is capable of crossing the blood–brain barrier and modulating microglial function, highlighting its relevance in gut–brain axis signaling (Li et al., 2019).

#### 3.4.5 Metabolic pathways and metabolites

The included studies show that MCI patients present alterations in aromatic amino acid metabolism (phenylalanine, tyrosine, tryptophan) (Table 3) that are key to neurotransmitter production (Hung et al., 2023; Kim et al., 2023). For instance, *Ruminococcus* participates in the tryptophan metabolism, a serotonin precursor, which is involved in mood regulation. Its reduction has been correlated with more severe affective symptoms in aMCI+ (Hung et al., 2023). In the same way, the MCI patients’ microbiota show reduction in the branched-chain amino acid catabolism pathways (valine, leucine, isoleucine), that participate in neurotransmitter synthesis and neuronal signaling. This highlights the role of metabolic microbiota dysfunction in early stages (Fan et al., 2025; Kim et al., 2023).

**Table 3:** Potentially altered metabolic pathways in MCI.

Category	Event	Effect	Reference
Aromatic amino acid metabolism: reduction in the biosynthesis pathways of phenylalanine, tyrosine, and tryptophan	Decreased production of serotonin and tryptamine, whose precursor is tryptophan.	Reduction in the availability of neurotransmitters, hormones, and immunomodulators	Chaudhari et al. (2023), Kim et al. (2023), Troci et al. (2024), and Zhao et al. (2025)
Branched-chain amino acid metabolism: reduction in the catabolic pathways of (Isoleucine, leucine, valine)	Decreased production of serotonin and norepinephrine	Reduction in the availability of neurotransmitters, hormones, and immunomodulators	Fan et al. (2025); Kim et al. (2023), and Li et al. (2019)
TCA reduction	Limited biosynthesis of essential metabolic precursors	Reduction of gut microbiome homeostasis	Chaudhari et al. (2023) and Kim et al. (2023)
Oxidative stress: Increased dysbiosis/permeability and LPS accumulation	Translocation of bacterial toxins from the intestine to the bloodstream and reach the brain	Induction of neuroinflammation	Kim et al. (2023), Li et al. (2019), Liu et al. (2021), and Troci et al. (2024)

Other affected metabolic pathways include the Krebs cycle, suggesting less efficient microbial energy metabolism, together with pathways linked to inflammation and neurodegeneration (Kim et al., 2023; Troci et al., 2024), and a decrease in nucleotide biosynthesis, especially UMP, is also observed, which can impact basic cellular processes (Chaudhari et al., 2023).

#### 3.4.6 Neuroinflammation

Gut dysbiosis is associated with pathological processes typical of neurodegenerative diseases (Kim et al., 2023). Microbial and metabolic changes correlate with Alzheimer’s biomarkers (p-tau, Aβ42), with systemic inflammatory states, and with mechanisms of synaptic dysfunction (Hung et al., 2023; Troci et al., 2024). In the same way, there is evidence about the correlation between microbial composition and brain function assessed by functional magnetic resonance imaging (fMRI) (Liu et al., 2021), which has described regions linked to memory and executive functions altered by microbiota assemblies (Liu et al., 2021). Furthermore, associations between microbial profiles and biomarkers such as p-tau/Aβ42 and C-reactive protein (CRP) are described, linking intestinal dysbiosis with neurodegenerative processes (Troci et al., 2024).

Some Gram-negative bacteria, such as *Porphyromonadaceae, Bacteroides* or *Proteobacteria*, contain pro-inflammatory LPS that can cross the intestinal barrier and the blood–brain barrier (BBB), promoting inflammatory processes and contributing to the pathology of AD (Troci et al., 2024). For instance, some molecules from *Escherichia coli* co-localize with amyloid plaques in the post-mortem brain tissue of patients with AD, suggesting that amyloid pathogenesis could be triggered by changes in the microbiome during MCI (Li et al., 2019).

## 4 Discussion

The relationship between MCI and gut microbiome is a critical area of study because microbial composition is affected not only by health, but also by intrinsic host factors (Auclair-Ouellet et al., 2025; Fan et al., 2025; Kim et al., 2023; Troci et al., 2024; Zhao et al., 2025). The research in MCI, AD, and PD has identified some demographic and lifestyle variables as important factors that influence the gut microbiome composition (Forero-Rodríguez et al., 2024; Li et al., 2024). The main variables described are age, comorbidities, diet, educational level, ethnicity and sex (Auclair-Ouellet et al., 2025; Bedarf et al., 2017; Chaudhari et al., 2023; Liang et al., 2022; Nuzum et al., 2023). However, the available evidence emphasizes the need for large-scale longitudinal studies conducted in well-characterized populations to validate these findings, given the substantial biological and methodological variability across studies (Guo et al., 2021; Hung et al., 2023; Kim et al., 2023). Consequently, findings from one population should not be directly extrapolated to others without appropriate validation (Fan et al., 2025).

The heterogeneity in the publications highlights that the changes observed in the microbiome of MCI patients are closely linked with the individual’s context, and strict control of these factors is indispensable for identifying robust disease-specific microbial biomarkers (Aho et al., 2024; Forero-Rodríguez et al., 2024; Ma, 2020; Stadlbauer et al., 2020). Studies consistently show that microbial alterations begin in the MCI stage with patterns very similar to those in AD, highlighting the promise of the microbiome for early diagnosis (Guo et al., 2021; Kim et al., 2023; B. Li et al., 2019). Furthermore, the potential use of the microbiome extends to other neurodegenerative diseases such as Amyotrophic Lateral Sclerosis (ALS) and Huntington’s disease (HD) (Auclair-Ouellet et al., 2025; Ekwudo et al., 2025; Zeng et al., 2020). In this sense, the alterations in microbiome composition and metabolism can be detected long before the onset of clinical dementia, even in preclinical stages of different neurodegenerative diseases (Auclair-Ouellet et al., 2025; Keshavarzian et al., 2015; Nagpal et al., 2019; Zeng et al., 2020; Zhang et al., 2021; Zhu et al., 2022).

For example, taxonomic alterations have been hypothesized to influence SCFA production in patients with AD and MCI, and are considered part of the same progressive event (Cabrera et al., 2025; Li et al., 2024; Li et al., 2023; Sheng et al., 2021). Another example is the widespread failure in the functional biosynthesis of amino acids by the MCI patient’s microbiota (Kim et al., 2023; J. Li et al., 2023). This is of high importance considering that some of these metabolites also present alterations in other neurodegenerative pathologies such as ALS, HD, and PD (Auclair-Ouellet et al., 2025; Fan et al., 2025; Hall et al., 2023; Keshavarzian et al., 2015; Nagpal et al., 2019; Nuzum et al., 2023; Rykalo et al., 2024; Zeng et al., 2020).

The main alterations in the gut microbiome observed in MCI, or amnestic mild cognitive impairment (aMCI) patients, are taxonomic and functionally associated with specific bacterial taxa abundance, although overall microbial diversity (alpha and beta) often shows no significant differences between patients with MCI and healthy controls (Kim et al., 2023; Liu et al., 2021). These alterations include a reduction in the abundance of potentially protective bacteria and an increase in the pro-inflammatory taxa abundance (Guo et al., 2021; Sheng et al., 2021). This pattern (proinflammatory dysbiosis) is considered an intermediate stage in the progression toward AD (Zhu et al., 2022).

Taking into account that these alterations occur before dementia, these specific microbial signatures may represent potential biomarkers pending longitudinal validation for early detection and identification of individuals at high risk of cognitive decline (Li et al., 2023; Liu et al., 2021; Zhu et al., 2022), even any authors have proposed that microbiome is a therapeutic target that can be modulated, through diet or probiotics, (Sheng et al., 2021; Zhang et al., 2021) and in this way to restore balance, and potentially prevent delay, or manage the symptoms of neurodegenerative and neuropsychiatric diseases (Auclair-Ouellet et al., 2025; Hall et al., 2023; Nagpal et al., 2020; Rykalo et al., 2024; Stadlbauer et al., 2020).

The heterogeneity across studies investigating the gut microbiota MCI represents a major limitation to the generalizability of findings and the identification of potential universal microbial biomarkers (Fan et al., 2025; Kim et al., 2023). This variability is attributed to a complex interplay of biological and methodological factors. On the one hand, variables such as ethnicity, geographic location, and dietary habits act as major modulators of the gut microbiome, generating distinct taxonomic profiles even among populations from the same geographic region (Guo et al., 2021; Kim et al., 2023; Liu et al., 2021). On the other hand, disparities in diagnostic criteria and the use of different sequencing technologies, bioinformatics pipelines, and taxonomic databases introduce substantial technical biases, further contributing to the variability observed across studies (Fan et al., 2025; Franyer et al., 2025; Kim et al., 2023). These limitations may lead to inaccurate estimates of the true microbial diversity and to the identification of associations that may not be reproducible across different populations and environmental settings.

## 5 Conclusion

In conclusion, the findings demonstrate that the gut microbiome is correlated with cognitive decline, and its altered composition provides clues for identifying high-risk populations and novel therapeutic targets in the early stages of the disease. Changes in microbiome composition alter its functional profile and, consequently, the range of metabolites and bioactive compounds supplied to the host. MCI is a progressive, self-reinforcing pathophysiological process that originates from the loss of intestinal homeostasis. The findings raise the possibility that MCI is a progressive, self-reinforcing pathophysiological process that may originate from the loss of intestinal homeostasis. This sequence begins with the degradation of the intestinal barrier and leads to increased intestinal permeability, allowing the translocation of bacterial toxins. These toxins cross the blood–brain barrier, triggering chronic neuroinflammation, oxidative stress, and alterations in the Krebs cycle tricarboxylic acid cycle (TCA). Simultaneously, disruptions in essential amino acid metabolism reduce the synthesis of key neurotransmitters such as serotonin, linking cognitive impairment to affective symptoms and accelerating brain atrophy.

In summary, evidence supports that gut microbiome analysis, particularly the assessment of specific taxa abundance, may serve as a non-invasive tool with high potential for early detection or prediction of the risk of MCI progression. For this purpose, gut metagenomics analysis is a high-resolution tool to identify robust biomarkers and develop predictive models for cognitive decline risk, which is fundamental for early detection and the development of intervention strategies for MCI.

In summary, the available evidence suggests that gut microbiome analysis, particularly the assessment of specific microbial taxa, may serve as a promising non-invasive approach for the early detection of MCI or the prediction of disease progression. In this context, gut metagenomic analysis offers high-resolution profiling that may facilitate the identification of robust microbial biomarkers and the development of predictive models for cognitive decline. However, the substantial biological and methodological heterogeneity across studies highlights the need for standardized approaches to study design, microbiome analysis, bioinformatic processing, and data interpretation to improve the reproducibility and comparability of future findings.

## Data Availability

The original contributions presented in the study are included in the article/Supplementary material, further inquiries can be directed to the corresponding author.
